# From Genetic Diversity to Genetic Gain: Molecular Approaches and Breeding Strategies in Tomato with Insights from Lithuanian Germplasm

**DOI:** 10.3390/ijms27125433

**Published:** 2026-06-16

**Authors:** Audrius Radzevičius, Danguolė Juškevičienė, Jonas Viškelis, Rasa Karklelienė

**Affiliations:** 1 Department of Vegetable Breeding and Technology, Lithuanian Research Centre for Agriculture and Forestry, Kauno 30, Kaunas Distr., 54333 Babtai, Lithuania; 2 Laboratory of Biochemistry and Technology, Lithuanian Research Centre for Agriculture and Forestry, Kauno 30, Kaunas District, 54333 Babtai, Lithuania

**Keywords:** marker-assisted selection, genomic prediction, genome editing, pangenomics, high-throughput phenotyping, fruit quality, carotenoids, regional adaptation

## Abstract

Tomato (*Solanum lycopersicum* L.) is a globally important vegetable crop and a major dietary source of bioactive compounds, including lycopene, ascorbic acid, phenolics, and minerals. Modern tomato breeding has substantially improved yield, uniformity, and postharvest performance; however, these gains have often been accompanied by reduced flavor quality, lower nutritional value, and narrowing of the genetic base. This review synthesizes available evidence on Lithuanian tomato germplasm and evaluates its relevance for future breeding strategies aimed at enhancing genetic gain under Northern European conditions. The review integrates published data on genetic diversity, molecular characterization, morphological traits, fruit quality parameters, and yield performance of Lithuanian cultivars and hybrids developed in Lithuania. SSR-based studies indicate moderate genetic diversity, with mean expected heterozygosity of approximately 0.51 and mean PIC values of 0.47 in cultivars and 0.45 in hybrids, while also confirming a relatively narrow breeding pool. Lithuanian cultivars display substantial variation in fruit morphology, dry matter, soluble solids, firmness, lycopene, ascorbic acid, and yield. Traditional cultivars such as ‘Svara’, ‘Milžinai’, ‘Slapukai’, and ‘Balčiai’ show valuable nutritional and technological traits, whereas hybrids such as ‘Auksiai H’, ‘Adas H’, and ‘Ainiai H’ demonstrate improved productivity and firmness. The available evidence suggests persistent yield–quality trade-offs, particularly between productivity, soluble solids content, antioxidant accumulation, and postharvest performance. Although Lithuanian germplasm does not represent exceptionally broad genetic diversity, it contains regionally adapted material with stabilized trait combinations useful for breeding resilience, nutritional quality, and adaptation to temperate environments. Future progress will require broadening the genetic base and integrating traditional breeding with marker-assisted selection, genomic selection, GWAS, genome editing, multi-omics, and pangenomic approaches. Overall, Lithuanian tomato germplasm represents a locally adapted regional resource for translating genetic diversity into genetic gain in modern tomato breeding.

## 1. Introduction

Tomato (*Solanum lycopersicum* L.) is one of the most widely cultivated vegetable crops worldwide and represents a major source of bioactive compounds in the human diet, including carotenoids, vitamins, phenolic compounds, and minerals [1,2,3]. Among these, lycopene and ascorbic acid are of particular importance due to their strong antioxidant properties and associated health benefits, such as reduced risk of cardiovascular diseases and certain cancers [4,5,6]. In addition to nutritional value, tomato fruit quality is determined by a complex interaction of morphological, biochemical, and technological traits, including fruit size, shape, firmness, soluble solids content, and dry matter [5,7,8,9].

In recent decades, tomato breeding has increasingly focused on improving yield, uniformity, and postharvest performance, often at the expense of flavor and nutritional quality [10,11,12]. That has been widely documented, particularly in commercial hybrid cultivars, where selection for agronomic performance may lead to reduced accumulation of sugars, organic acids, and secondary metabolites [13,14,15]. This trend has renewed interest in regional germplasm collections, particularly those developed under less intensive breeding systems where flavor and biochemical quality may have been maintained to a greater extent [16,17].

European tomato germplasm exhibits considerable diversity shaped by regional breeding programs, climatic conditions, and consumer preferences [18,19,20]. Nevertheless, several studies have demonstrated that modern cultivated tomatoes generally possess a relatively narrow genetic base compared to wild relatives, largely due to domestication bottlenecks and intensive selection [21,22,23]. This limited genetic diversity is particularly evident in regional breeding pools, where a restricted number of parental lines are used [24,25], and SSR-based studies consistently show that cultivated tomato possesses substantially lower allelic diversity than its wild relatives, reflecting the strong domestication bottleneck characteristic of the species [26,27,28].

Lithuanian tomato breeding, primarily conducted at the Institute of Horticulture of the Lithuanian Research Centre for Agriculture and Forestry (LRCAF IH), has produced a range of cultivars and hybrids adapted to Northern European climatic conditions. These cultivars are characterized by specific traits such as moderate yield stability, tolerance to fluctuating temperatures, and distinctive fruit quality attributes [29,30,31]. Previous studies on Lithuanian tomato germplasm have revealed moderate genetic diversity based on SSR markers, with polymorphism information content (*PIC*) values and heterozygosity levels comparable to those reported for other cultivated tomato groups [32,33,34]. Despite this, comprehensive synthesis of morphological, biochemical, and productivity traits of Lithuanian cultivars remains limited.

Fruit quality in tomatoes is strongly influenced by environmental conditions, particularly in temperate regions where lower light intensity and temperature can restrict the accumulation of sugars and carotenoids [35,36,37]. Nevertheless, local cultivars often exhibit stable performance and balanced biochemical composition under such conditions, making them valuable genetic resources for breeding programs targeting climate resilience and nutritional improvement [38,39,40]. Traits such as soluble solids content (°Brix), dry matter, and firmness are critical for both fresh consumption and processing, while bioactive compounds such as lycopene and vitamin C contribute to functional food value [41,42,43].

Recent advances in plant breeding emphasize the importance of integrating molecular, biochemical, and phenotypic data to develop improved cultivars [44,45,46,47,48]. In this context, the evaluation of national germplasm collections plays a key role in identifying promising genotypes that combine high yield with superior fruit quality [49,50,51,52,53,54]. Lithuanian vegetable cultivars, including traditional varieties and modern hybrids, provide a unique opportunity to assess such traits within a relatively narrow but distinct genetic background [13,29,30,31,32,55,56,57,58].

Despite the availability of individual studies on Lithuanian tomato breeding and fruit quality, existing research remains largely fragmented and predominantly descriptive, lacking an integrative and critical synthesis that connects genetic, biochemical, and agronomic traits within a broader European and global context. Such a synthesis is essential not only to summarize existing knowledge, but also to identify limitations in current research, evaluate breeding features, and position Lithuanian germplasm within global tomato breeding strategies. Despite the increasing availability of molecular tools in tomato breeding, the connection between genetic diversity and realized genetic gain remains insufficiently addressed, particularly in regionally adapted germplasm. Most studies on Lithuanian tomato cultivars are descriptive, focusing on phenotypic traits or limited molecular characterization, without explicitly linking genetic variation to breeding efficiency or long-term improvement potential.

Therefore, this review aims to synthesize current knowledge on Lithuanian tomato germplasm by integrating published evidence on genetic diversity, morphological and agronomic traits, fruit quality parameters, and yield performance, while highlighting its value for future breeding under Northern European conditions. A key challenge is not only to characterize diversity but to understand how it can be effectively translated into genetic gain through modern breeding strategies.

## 2. Review Methodology

This review was conducted using a structured literature synthesis approach to ensure broader coverage, greater transparency, and stronger critical analysis than a purely narrative summary. Information related to Lithuanian tomato cultivars and hybrids developed in Lithuania was collected and evaluated alongside relevant international literature on tomato genetic diversity, fruit quality, and breeding.

The review combined targeted database searches with manual screening of institutional publications and historical Lithuanian breeding literature. Much of this material is poorly represented in international indexing systems, complemented by institutional publications, dissertations, and printed scientific sources available in library collections. Search terms included combinations of the following keywords: *Solanum lycopersicum*; Lithuanian tomato germplasm; genetic diversity; SSR markers; fruit quality; lycopene; ascorbic acid; yield; breeding; and molecular breeding. To provide both historical and contemporary context, publications spanning early Lithuanian breeding reports to recent molecular and compositional studies were considered.

The literature identification, screening, eligibility assessment, and study selection process followed PRISMA guidelines and is summarized in the PRISMA flow diagram presented in Supplementary Figure S1.

Publications were included if they met one or more of the following criteria:

(1) reported data on Lithuanian tomato cultivar development;
(2) presented quantitative information on genetic diversity, morphology, biochemical composition, fruit quality, or yield performance of Lithuanian tomato germplasm;
(3) provided international comparative evidence necessary to interpret Lithuanian results within a broader breeding and physiological context. Studies lacking relevant quantitative or clearly interpretable qualitative information were not prioritized.

Where available, cultivar-level data were extracted and compared across studies, including plant growth type, fruit morphology, average fruit mass, dry matter, soluble solids, fruit firmness, lycopene content, ascorbic acid content, and yield. Because the reviewed studies differed in experimental design, cultivation system, season, and analytical methodology, the present review does not treat all values as directly equivalent. Instead, the evidence was interpreted comparatively and critically, with particular attention to recurring trait patterns, likely genotype–environment interactions, and consistent breeding limitations.

Thus, the purpose of the methodology was not only to gather Lithuanian data, but also to move beyond description by situating these data within the wider literature on tomato domestication, regional breeding systems, fruit-quality physiology, and modern breeding strategies.

The following sections critically discuss Lithuanian tomato germplasm in relation to molecular diversity, fruit quality traits, yield performance, and modern breeding strategies.

## 3. Lithuanian Tomato Germplasm in the Context of Modern Tomato Breeding

### 3.1. Genetic Resources and Molecular Characterization of Lithuanian Tomato Germplasm

The most comprehensive molecular characterization of Lithuanian tomato germplasm used seven SSR markers to assess 13 varieties and 6 hybrids developed at the Institute of Horticulture, Lithuanian Research Centre for Agriculture and Forestry (LRCAF IH). A total of 24 alleles were detected across the varieties and 26 alleles across the hybrids [32]. Expected heterozygosity (*He*) averaged 0.51 for both varieties and hybrids, while Polymorphism Information Content (*PIC*) values averaged 0.47 for varieties (range 0.13–0.68) and 0.45 for hybrids (range 0.31–0.61) (Table 1). The most informative SSR primers for varieties were TMS52, TGS0007, LEMDDNa, and Tom236-237. UPGMA cluster analysis demonstrated groupings consistent with known pedigree relationships; for example, ‘Viltis’ (parent of ‘Laukiai’) and ‘Aušriai’ (progeny of ‘Jurgiai’) clustered together. The Lithuanian varieties were all homozygous at tested loci, whereas hybrids were heterozygous at some loci, inheriting different alleles from parental forms. It was noted that the gene pool of Lithuanian tomato genotypes may be relatively narrow compared to other regions [32].

It should be noted that expected heterozygosity (*He*) is calculated from allele frequencies and reflects the probability that two randomly selected alleles differ within a population. Therefore, *He* is not equivalent to observed heterozygosity (*Ho*), which measures the actual proportion of heterozygous individuals. Although cultivars and hybrids exhibited identical average *He* values (0.51), cultivars were completely homozygous (*Ho* = 0), whereas hybrids showed an average observed heterozygosity of 0.34. This indicates that similar allele-frequency distributions were present in both groups despite clear differences in zygosity at the individual level.

The molecular diversity detected in Lithuanian tomato germplasm can be compared with diversity levels reported for other cultivated tomato collections. SSR studies conducted in European breeding programs, including Italian, Spanish, and broader international cultivated tomato collections, generally report *PIC* values between approximately 0.30 and 0.70 and 2–9 alleles per locus [17,18,26,27,28,33]. Wild tomato species consistently exhibit substantially higher allelic richness and genetic diversity than cultivated tomatoes because domestication and intensive breeding have reduced variation within commercial germplasm pools [21,22,23,26,27,28].

The average *PIC* value observed in Lithuanian cultivars (0.47) therefore indicates moderate marker informativeness and falls near the middle of the range typically reported for cultivated tomato germplasm rather than at its lower boundary. However, the relatively small number of alleles detected and the pedigree-based clustering pattern suggest a comparatively narrow breeding base, consistent with the long-term use of a limited number of parental lines within regional breeding programs.

Although the available SSR data confirm the distinctiveness and pedigree coherence of Lithuanian cultivars, they also indicate a need to broaden the breeding base and supplement classical marker approaches with higher-resolution genomic tools. SSR markers remain useful for cultivar identification and pedigree analysis, but they provide only a partial representation of genome-wide diversity and may underestimate structural genomic variation, including insertions, deletions, copy-number variation, and presence–absence variation [23,26,27,59,60,61].

Importantly, the available SSR dataset should be interpreted as a preliminary marker-based assessment rather than a comprehensive representation of genome-wide diversity. The use of only seven SSR loci provides limited resolution compared with modern high-density SNP genotyping, genotyping-by-sequencing, or whole-genome resequencing approaches [23,24,25,28,59,60]. Therefore, the current data are insufficient to detect most structural variants, rare alleles, locally adapted haplotypes, or genome-wide patterns of linkage disequilibrium. Future studies on Lithuanian tomato germplasm should prioritize genome-wide marker platforms and resequencing-based analyses to more accurately assess genetic diversity, identify breeding-relevant alleles, and support the transition from diversity description to genetic gain.

Consequently, the current understanding of Lithuanian tomato germplasm diversity remains incomplete and probably underestimates the extent of available allelic and structural variation.

The original characterization study also compared molecular and phenotypic relationships among Lithuanian tomato genotypes. In that study, UPGMA clustering based on seven SSR markers was compared with clustering based on twelve morphological traits using a tanglegram analysis [32]. The results showed only partial correspondence between molecular and morphological groupings, indicating that phenotypic similarity does not always reflect underlying genetic similarity [32,33,34]. This supports the need to combine phenotypic evaluation with molecular fingerprinting when selecting parental material for breeding [32,44,45,46,47,48]. However, the available SSR dataset remains insufficient for robust marker–trait association analysis because only seven SSR loci were evaluated and genome-wide marker coverage was not available. Therefore, the current evidence should be interpreted as a preliminary integration of molecular and phenotypic diversity rather than as a formal association analysis. Future studies should combine standardized multi-environment phenotyping with high-density SNP genotyping or whole-genome resequencing to identify marker–trait associations related to fruit quality, yield stability, firmness, disease resistance, and adaptation to northern European conditions [59,60,61,62,63,64,65,66].

At present, the transition from genetic diversity to genetic gain in Lithuanian tomato germplasm should be viewed as a strategic breeding framework rather than as a fully resolved marker–trait prediction system.

### 3.2. Marker-Assisted Selection, GWAS and QTL Mapping

Marker-assisted selection remains one of the most important traditional molecular approaches in tomato breeding (Table 2). The main advantage of MAS lies in reducing the need for late-stage phenotypic selection, particularly for traits with relatively simple genetic architecture [44,45,46,47,48,49,50,51]. This is especially useful for traits controlled by major genes, such as disease resistance, plant architecture, fruit shape, and some fruit-quality traits [59,60,61,62,63,64,65,66,67,68,69]. Disease resistance represents one of the most successful applications of marker-assisted selection in tomato breeding because numerous resistance genes have been identified and are routinely used in breeding programs. Major resistance genes include *Tm-2^2^* for resistance to Tomato mosaic virus (*ToMV*), *Ve1* for Verticillium wilt, *I*, *I-2*, and *I-3* for Fusarium wilt, *Mi-1.2* for root-knot nematodes, *Ph-3* for late blight, *Ty-1/Ty-3* and *Ty-2* for Tomato yellow leaf curl virus (*TYLCV*), and *Sw-5b* for Tomato spotted wilt virus (*TSWV*). Molecular markers linked to these genes enable early and reliable selection of resistant genotypes and facilitate the pyramiding of multiple resistance genes into a single cultivar, thereby improving the durability and breadth of disease resistance [64,65,66].

Beyond disease resistance, marker-assisted selection is also widely applied to genes controlling fruit morphology, plant architecture, ripening, and fruit quality traits, which represent important breeding targets in modern tomato improvement programs. In tomato, several major genes and QTLs are central to breeding. *FW2.2* is associated with fruit size and plays a major role in domestication-related fruit enlargement [52]. *SUN* and *OVATE* influence fruit shape by modifying cell division and fruit elongation patterns [50,51]. *SELF-PRUNING* (*SP*) regulates determinate growth habit and is highly relevant for production system adaptation [49,50,51,52]. Ripening-related genes such as *RIN*, *NOR*, and *CNR* regulate fruit maturation, texture, shelf life, and postharvest quality [11,67,70,71]. Genes involved in carotenoid biosynthesis, including *PSY1* and *LCY-B* cyclases, are important for nutritional quality and lycopene accumulation [64,65,66,67].

Although MAS is useful, it is less efficient for highly polygenic traits such as yield, soluble solids, flavor, stress tolerance, and adaptation. Therefore, MAS should be viewed as part of a broader breeding strategy rather than a complete solution.

For Lithuanian tomato germplasm, disease-resistance loci and major quality-related genes represent particularly valuable targets for future breeding programs. The relatively narrow diversity detected using a limited SSR marker set suggests that marker-assisted introgression of resistance genes and favorable quality alleles from broader germplasm pools could help expand the breeding base while maintaining the local adaptation, fruit quality, and productivity characteristics that distinguish Lithuanian cultivars. In this context, MAS provides a practical bridge between the existing genetic resources and more advanced genomic breeding approaches.

Genome-wide association studies and QTL mapping provide tools for identifying genomic regions associated with agronomic and fruit-quality traits. In tomato, these approaches have been used to dissect complex traits such as fruit weight, shape, soluble solids, carotenoid content, flavor volatiles, and disease resistance [7,18,23,24,25]. GWAS is particularly useful when combined with diverse germplasm collections, while biparental QTL mapping remains effective for traits segregating in specific breeding populations [59,60].

The loci and candidate genes identified through GWAS and QTL mapping provide the foundation for developing molecular markers subsequently used in marker-assisted selection programs. Therefore, GWAS, QTL mapping, and MAS should be considered complementary components of a unified molecular breeding framework rather than independent approaches.

Lithuanian tomato germplasm has not yet been fully analyzed using high-density SNP genotyping or GWAS. This represents an important limitation but also an opportunity. Future studies should combine Lithuanian cultivars with broader European and global collections to identify whether local adaptation and quality traits are associated with unique alleles or allele combinations.

### 3.3. Emerging Molecular Approaches for Enhancing Genetic Gain

#### 3.3.1. Genomic Selection, Pangenomics and Broadening the Breeding Base

Genomic selection has substantially advanced modern plant breeding because it uses genome-wide marker data to predict genomic estimated breeding values. Unlike MAS, which focuses on selected major loci, genomic selection captures the cumulative effects of many small-effect loci distributed across the genome. This distinction becomes especially relevant for traits such as yield stability or flavor, where phenotypic expression depends on the cumulative contribution of numerous small-effect loci [44,45,46,59,60,61].

For tomato breeding, genomic selection can enhance genetic gain by increasing selection accuracy, enabling earlier selection, reducing generation interval, and supporting multi-trait selection [62,63]. That is important for regional germplasm pools such as Lithuanian tomato cultivars, where the number of available genotypes may be limited and phenotyping across environments is expensive.

The predictive ability of genomic selection can be further enhanced by incorporating broader sources of genomic variation. In this context, pangenomic approaches have become increasingly important because a single reference genome cannot capture all structural variation present within a species. Tomato pangenomes reveal genes, alleles, and structural variants that may be absent from the reference genome but are present in wild relatives, landraces, and regional germplasm collections [59,60,61,69].

Such information can improve marker density, increase the representation of functional variation in prediction models, and facilitate the identification of favorable alleles associated with adaptation, disease resistance, fruit quality, and stress tolerance. Therefore, pangenomics and genomic selection should be viewed as complementary approaches within a unified framework for accelerating genetic gain and broadening the breeding base.

For Lithuanian tomato breeding, pangenomic resources could help identify missing diversity and guide the strategic introgression of novel alleles from broader germplasm sources while preserving locally adapted trait combinations. In the Lithuanian breeding context, genomic selection and pangenomics should be directly linked to several practical bottlenecks: improving yield stability under short growing seasons, maintaining fruit quality under low-light conditions, broadening the narrow SSR-defined genetic base, and combining locally adapted phenotypes with novel alleles for disease resistance, firmness, and nutritional quality. Genomic prediction models could be especially useful for complex traits such as yield stability, soluble solids, flavor-related traits, and adaptation to fluctuating temperature and light regimes, which are difficult to improve using single-marker selection alone.

#### 3.3.2. Genome Editing

Genome editing, particularly CRISPR/Cas technology, provides a powerful tool for precise modification of target genes. In tomato, genome editing has been used to modify plant architecture, fruit size, ripening, nutritional traits, and stress responses. For traits controlled by known genes, genome editing can accelerate genetic gain by directly introducing favorable allelic variation without multiple generations of backcrossing [59,60,61].

The regulatory landscape for genome editing in Europe is currently undergoing substantial change. In 2023, the European Commission proposed a new regulatory framework for plants obtained through certain new genomic techniques (NGTs) (COM(2023)411), aiming to distinguish between plants that could also occur naturally or through conventional breeding and those requiring more extensive regulatory oversight. Recent progress in the legislative process indicates that certain categories of genome-edited plants may be regulated separately from conventional genetically modified organisms, potentially facilitating their future use in European breeding programs [72,73]. Beyond its direct breeding applications, genome editing remains an important research tool for validating gene function, dissecting complex traits, and identifying targets for marker-assisted and genomic selection. For Lithuanian germplasm, genome editing should be considered primarily as a functional validation and trait-targeting tool rather than as a stand-alone breeding solution. Candidate applications include testing genes associated with determinate growth habit and adaptation to short growing seasons, cell-wall metabolism and fruit firmness, carotenoid biosynthesis and lycopene accumulation, and stress-response pathways related to low temperature and reduced light availability [59,60,61,67,70,71]. Such targeted validation would help identify alleles that could subsequently be used in conventional crossing, marker-assisted selection, or genomic selection schemes [44,45,46,59,60,61,62].

#### 3.3.3. Multi-Omics and Systems Breeding

Attempts to explain tomato fruit quality using only genomic information have proven insufficient because most quality traits emerge from highly interconnected metabolic and developmental processes. Therefore, genomics alone is insufficient to fully explain variation in flavor, carotenoids, vitamin C, soluble solids, and texture. Integration of transcriptomics, metabolomics, proteomics, and phenomics provides a systems-level understanding of trait regulation. For example, lycopene accumulation depends on carotenoid biosynthesis, chloroplast-to-chromoplast differentiation, fruit ripening regulation, and environmental factors such as light and temperature. Similarly, soluble solids depend on photosynthesis, assimilate transport, sugar metabolism, water relations, and fruit sink strength. Multi-omics approaches can therefore help identify the molecular mechanisms underlying yield stability, fruit quality, flavor development, firmness, carotenoid accumulation, and adaptation to low-temperature and low-light conditions. For Lithuanian tomato germplasm, these approaches are particularly relevant because breeding objectives extend beyond yield improvement to include fruit quality, nutritional value, disease resistance, and adaptation to northern European environments [59,60,61,62].

#### 3.3.4. High-Throughput Phenotyping and Digital Agriculture

While advances in genomics have accelerated the identification of genes and markers associated with important agronomic traits, phenotyping remains a critical component of modern breeding programs. Accurate characterization of plant performance is essential for linking genotype to phenotype and maximizing the effectiveness of genomic selection, genome-wide association studies, and other molecular breeding approaches [74,75].

Recent developments in high-throughput phenotyping (HTP) enable the rapid collection of large-scale phenotypic data using imaging and sensor-based technologies. Digital imaging systems can quantify fruit size, shape, color, canopy architecture, growth dynamics, and disease symptoms with greater precision and throughput than conventional visual assessment. In tomato, image-based phenotyping has been successfully applied to fruit morphology analysis, ripening assessment, yield prediction, and stress-response evaluation [74,75,76].

Spectral sensing technologies, including multispectral and hyperspectral imaging, provide additional opportunities for non-destructive assessment of physiological and biochemical traits. These approaches can estimate chlorophyll content, photosynthetic activity, water status, carotenoid accumulation, and early disease development before visible symptoms appear [76,77]. Combined with machine-learning approaches, such datasets can improve the prediction of complex traits and enhance breeding efficiency [75,78].

For Lithuanian tomato germplasm, the integration of digital imaging, spectral sensing, molecular markers, and environmental monitoring could facilitate the identification of genotypes with superior fruit quality, disease resistance, stress tolerance, and adaptation to northern European growing conditions. Consequently, high-throughput phenotyping is expected to become an increasingly important component of future tomato breeding strategies.

### 3.4. Fruit Quality Traits and Phenotypic Diversity of Lithuanian Tomato Cultivars

#### 3.4.1. Morphological Diversity of Lithuanian Tomato Cultivars

Across the reviewed studies, Lithuanian tomato cultivars and hybrids exhibited substantial diversity in plant growth type and fruit morphology (Table 3). Both determinant and indeterminate genotypes were represented, reflecting breeding efforts targeting different production systems. Determinant cultivars such as ‘Aušriai’, ‘Viltis’, ‘Slapukai’, ‘Svara’, and ‘Balčiai’ are typically associated with concentrated fruit set and suitability for open-field cultivation, whereas indeterminate genotypes (e.g., ‘Milžinai’, ‘Skariai’, ‘Rutuliai’) are more suitable for greenhouse production due to continuous growth and extended harvesting periods.

This clear differentiation between growth types demonstrates the genetic control of shoot determinacy, primarily regulated by genes such as *SELF-PRUNING (SP)*, which determines the transition from vegetative to reproductive growth [49]. Determinate genotypes are characterized by synchronized fruit set and reduced vegetative growth, making them particularly suitable for mechanized or open-field production systems. In contrast, indeterminate genotypes maintain continuous meristem activity, allowing prolonged flowering and fruiting, which is advantageous under protected cultivation conditions such as greenhouses [7,13].

Fruit morphology in Lithuanian cultivars was predominantly slightly flattened, a trait commonly associated with traditional European germplasm, which is also traditional for Lithuanian cultivars. However, other shapes, including round (‘Svara’, ‘Auksiai H’, ‘Adas H’), cylindrical (‘Skariai’), and obovoid (‘Ainiai H’), were also observed. The presence of round, cylindrical, and obovoid fruit types reflects increasing diversification driven by market demand and breeding objectives. Fruit shape is a complex trait controlled by multiple quantitative trait loci (QTLs), including *SUN*, *OVATE*, and FASCIATED, which regulate cell division patterns and fruit elongation [50,51]. The diversification observed in Lithuanian germplasm therefore represents both genetic variation and targeted selection for market-oriented traits, such as uniformity, visual appeal, and processing efficiency.

Beyond visual appearance, fruit morphology directly affects packaging efficiency, market segmentation, and suitability for industrial processing; for example, round and uniform fruits are generally preferred in fresh markets due to their aesthetic quality, while elongated or cylindrical shapes may be advantageous for processing due to improved slicing and packing characteristics [18,20]. The coexistence of multiple fruit types within Lithuanian cultivars suggests a broadening of breeding targets beyond traditional local types, aligning with global trends in tomato market segmentation.

Fruit weight variation among genotypes (35–161 g) further illustrates the diversity of breeding strategies. Large-fruited cultivars such as ‘Milžinai’ and ‘Skariai’ are typically associated with fresh consumption and higher consumer appeal, whereas small-fruited hybrids (e.g., ‘Auksiai H’, ‘Adas H’) are increasingly important in niche markets such as cherry and snack tomatoes. Fruit size is a polygenic trait influenced by loci such as *FW2.2*, which regulates cell division during early fruit development [52]. The wide range of fruit weights observed in Lithuanian germplasm therefore reveals both genetic diversity and selection for specific market classes. The observed morphological diversity suggests a transition from traditional, locally adapted cultivars toward more diversified and market-oriented breeding outputs. Maintaining a broader range of breeding targets is increasingly important under modern horticultural conditions but should be balanced with the preservation of locally adapted traits and genetic resources. In this respect, Lithuanian germplasm appears to combine both continuity with traditional breeding history and gradual adaptation to changing market expectations.

#### 3.4.2. Dry Matter and Soluble Solids Content

Dry matter content is a key determinant of tomato fruit quality, as it integrates the accumulation of sugars, organic acids, and structural components that collectively influence flavor intensity, nutritional value, and processing performance. Published data for Lithuanian tomato cultivars indicate a dry matter content ranging from 4.64% to 8.10% [13,14,36]. For comparison, tomato cultivars grown under temperate climatic conditions in Europe and other northern production regions typically contain approximately 4–8% dry matter, depending on genotype, cultivation system, and environmental conditions. Thus, the values reported for Lithuanian cultivars fall within the upper part of the range commonly observed under temperate growing conditions, with several cultivars exceeding 7%, which is generally considered favorable for both fresh-market quality and processing applications [36,54,79,80]. Higher dry matter content is generally associated with improved processing suitability, particularly for paste and sauce production, due to increased solid yield and reduced energy requirements during concentration [53].

Soluble solids content (°Brix), which primarily illustrates the concentration of soluble sugars such as glucose and fructose, is one of the most important determinants of tomato flavor and consumer acceptance [7,10]. The observed range (4.32–6.5 °Brix) falls within typical limits reported for greenhouse-grown tomatoes in Northern Europe [36,54]. The relatively high values recorded in cultivars such as ‘Slapukai’ and ‘Adas H’ indicate enhanced sugar accumulation and suggest superior sensory quality, as sweetness is a major contributor to flavor perception. Environmental factors also play a critical role in determining dry matter and soluble solids content. In Northern European conditions, reduced solar radiation and lower temperatures can limit photosynthetic activity and carbohydrate accumulation, resulting in moderate but relatively stable °Brix values across cultivars [35,36]. The stability observed in Lithuanian cultivars suggests a high degree of adaptation to local climatic conditions, where genotypes are selected for consistent performance rather than extreme quality traits.

Importantly, several cultivars, including ‘Aušriai’, ‘Laukiai’, and ‘Balčiai’, demonstrated balanced combinations of dry matter and soluble solids, indicating their suitability for both fresh consumption and processing applications. Such balance is particularly valuable for breeders, as it suggests the possibility of combining acceptable yield with desirable flavor and processing characteristics. Such combinations are difficult to achieve because increasing sugar accumulation frequently occurs at the expense of fruit size or total yield [7,10].

The observed variation in dry matter and soluble solids content is consistent with the complex interaction between genetic factors, environmental conditions, breeding objectives, and it also illustrates one of the central challenges of tomato improvement under temperate conditions: increasing sugar accumulation without compromising yield. From a critical perspective, Lithuanian germplasm appears to offer relatively stable and balanced quality profiles, but not necessarily extreme sweetness or solids concentration. This suggests that, compared with germplasm developed under higher-light environments, Lithuanian breeding has favored stability and adaptation over maximum quality expression. Future progress will therefore require more targeted integration of physiology, genetics, and cultivation practices [7,10,13,14,55,56,57,58].

#### 3.4.3. Fruit Firmness and Postharvest Quality

Fruit firmness is a key technological trait affecting transportability, shelf life, and resistance to mechanical damage [70,71]. Across the reviewed studies, firmness ranged from 12.5 N cm^−2^ (‘Skariai’) to 22.9 N cm^−2^ (‘Balčiai’), demonstrating considerable variability among genotypes. Cultivars such as ‘Balčiai’, ‘Adas H’, and ‘Auksiai H’ exhibited higher firmness values (>19 N cm^−2^), suggesting good postharvest performance and suitability for long-distance transportation. In contrast, softer fruits (e.g., ‘Skariai’, ‘Dručiai’) may be more prone to mechanical damage but could offer superior sensory qualities, as softer texture is often associated with better eating quality [7,11,13].

The contrast between cultivars such as ‘Balčiai’ and ‘Skariai’ illustrates the classical breeding dilemma between postharvest durability and eating quality. Selection for increased firmness must be balanced with the preservation of desirable organoleptic properties [67,71].

This relationship is especially relevant in regional breeding systems, where local acceptance may depend not only on appearance and storability, but also on flavor and texture characteristics valued by consumers. Lithuanian germplasm appears to include material at both ends of this spectrum, suggesting that it could support breeding both for postharvest performance and for improved eating quality, depending on breeding priorities.

#### 3.4.4. Lycopene Content

Lycopene is the dominant carotenoid in tomato fruits and a major determinant of their nutritional value due to its strong antioxidant properties [4,6]. Reported values of lycopene content ranged from 38.4 (‘Auksiai H’) to 95 mg kg^−1^ (‘Svara’), indicating significant genetic variability. High lycopene concentrations were observed in traditional cultivars such as ‘Svara’, ‘Milžinai’, ‘Laukiai’, and ‘Viltis’, suggesting that these genotypes retain strong nutritional characteristics. In contrast, hybrid cultivars (‘Auksiai H’, ‘Adas H’) exhibited lower lycopene levels, which may reflect breeding priorities focused on yield, uniformity, and shelf life rather than nutritional composition, a trend widely reported in modern tomato breeding [12,13].

The relatively high lycopene content observed in several Lithuanian cultivars is notable when compared to typical values reported for commercial tomatoes in Northern Europe, where climatic conditions such as lower light intensity and temperature often limit carotenoid accumulation [35,37]. This proves that local germplasm can represent a donor material in future breeding programs aiming to improve functional food properties and enhance antioxidant content in tomato fruits [6,41].

The contrast between traditional cultivars and hybrids suggests that traditional or less intensively optimized breeding material may retain useful nutritional traits lost or diluted in highly productivity-oriented germplasm. In Lithuanian tomatoes, lycopene appears to be one of the clearest traits differentiating traditional cultivars from modern hybrids. This pattern is consistent with the frequently reported yield–quality trade-off and indicates that local cultivars may serve as useful donor material for improving nutritional quality in future breeding programs.

#### 3.4.5. Ascorbic Acid Content

Ascorbic acid content among the evaluated Lithuanian tomato cultivars and hybrids showed considerable variation, ranging from 7.8 to 19.3 mg 100 g^−1^ fresh weight (FW) (Table 3). Such variability is typical for tomato fruits and confirms both genetic and environmental influences on vitamin C accumulation [13,41,42]. The highest concentrations were recorded in ‘Balčiai’ (19.3 mg 100 g^−1^ FW) and ‘Slapukai’ (19.0 mg 100 g^−1^ FW), followed by hybrid genotypes such as ‘Ainiai H’ (17.8 mg 100 g^−1^ FW), ‘Auksiai H’ (17.6 mg 100 g^−1^ FW), and ‘Adas H’ (17.4 mg 100 g^−1^ FW). These values indicate strong nutritional potential and suggest that both traditional cultivars and hybrids can accumulate substantial amounts of vitamin C, as previously reported for diverse tomato germplasm [41,42,43].

In contrast, lower ascorbic acid levels were observed in ‘Viltis’ (7.8 mg 100 g^−1^ FW), ‘Rutuliai’ (7.8 mg 100 g^−1^ FW), and ‘Milžinai’ (7.9 mg 100 g^−1^ FW), reflecting genotype-dependent differences in antioxidant accumulation. Intermediate values were found in cultivars such as ‘Aušriai’ (14.1 mg 100 g^−1^ FW), ‘Laukiai’ (12.2 mg 100 g^−1^ FW), and ‘Svara’ (10.9 mg 100 g^−1^ FW).

Generally, the observed range falls within the typical limits reported for tomatoes grown under temperate climatic conditions, where environmental factors such as light intensity, temperature, and cultivation practices strongly influence ascorbic acid biosynthesis [35,36]. Considerable genotypic variation in ascorbic acid content indicates strong genetic control of this trait and supports its potential use in quality-oriented breeding programs [41,43]. In contrast to lycopene, ascorbic acid does not follow a simple cultivar-versus-hybrid pattern. Instead, the observed variation can be associated with stronger genotype × environment interactions and more complex metabolic regulation.

#### 3.4.6. Integrated Evaluation of Cultivar Performance

When considering all traits together, several cultivars demonstrated particularly favorable combinations of morphological and biochemical characteristics. For example, ‘Svara’ combined high lycopene content (95 mg kg^−1^), high firmness (18.2 N cm^−2^), and good soluble solids, indicating excellent quality. ‘Slapukai’ exhibited the highest soluble solids (6.5 °Brix) and high dry matter (7.5%), suggesting superior flavor. ‘Milžinai’ showed very large fruit size and high lycopene content, making it attractive for fresh consumption. ‘Balčiai’ demonstrated exceptional firmness (22.9 N cm^−2^), indicating strong postharvest potential. Cultivars combining acceptable firmness with elevated soluble solids and antioxidant content remain especially attractive because these traits are often negatively associated [7,11]. The coexistence of high soluble solids, firmness, and antioxidant content in certain cultivars suggests that favorable trait combinations can be achieved despite commonly reported trade-offs between yield, flavor, and postharvest characteristics [12,13,14]. This reviewed evidence highlights the potential of Lithuanian tomato cultivars as valuable genetic resources for breeding programs targeting improved fruit quality, nutritional value, and adaptation to Northern European growing conditions.

These integrated comparisons should also be interpreted with caution. The reviewed datasets originate from different studies and may reflect variation in cultivation system, season, maturity stage, and analytical methodology; while cross-study synthesis is useful for identifying broad trait patterns, direct cultivar ranking across all traits should not be overinterpreted as if all genotypes had been evaluated under a single uniform experimental design. This limitation is important and has often been underemphasized in descriptive summaries. Similar conclusions regarding the importance of locally adapted germplasm for developing resilient and high-quality cultivars under specific environmental conditions have been reported elsewhere [38,47].

#### 3.4.7. Breeding Implications and Future Perspectives

The observed diversity in fruit quality traits represents both traditional selection and modern hybrid breeding strategies. Lithuanian cultivars maintain relatively high nutritional quality, particularly in terms of lycopene content, while hybrids tend to emphasize technological traits such as firmness and uniformity, a trend widely reported in tomato breeding programs [12,13].

Future breeding efforts should aim to combine these attributes by increasing soluble solids without compromising yield, maintaining high lycopene content in hybrid lines, and improving firmness while preserving sensory quality. These objectives align with current strategies focused on overcoming the well-known interactions between yield, flavor, and postharvest performance through integrated genetic and physiological approaches [7,10,14].

**Figure 1:**
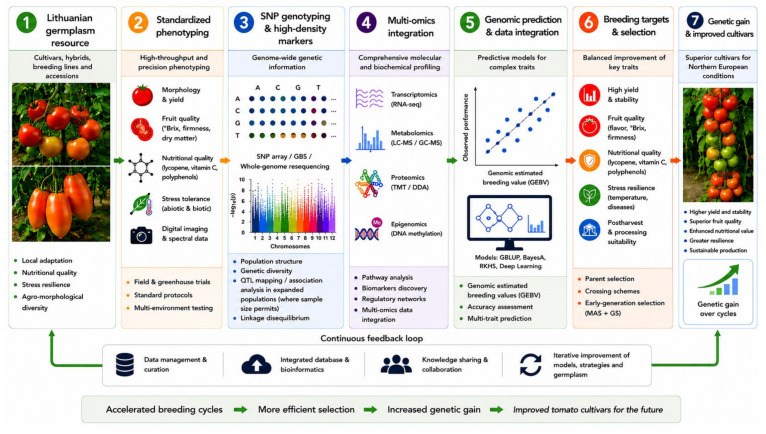
Integrated molecular breeding framework for enhancing genetic gain in tomato using Lithuanian germplasm.

From a critical breeding perspective, Lithuanian germplasm appears especially valuable not because it represents exceptionally broad diversity, but because it contains locally adapted material in which useful trait combinations have already been stabilized under temperate conditions. This makes it relevant for breeding strategies aimed at resilience, regional adaptation, and nutritional quality (Figure 1). At the same time, the narrow gene pool and moderate molecular diversity suggest that future breeding progress may increasingly depend on combining Lithuanian material with broader germplasm resources and on adopting modern genomic tools to accelerate selection.

These integrated strategies may facilitate the development of tomato cultivars adapted to changing climatic conditions and evolving consumer demands, particularly in temperate regions where environmental constraints further influence fruit quality [35,38].

### 3.5. Yield Performance and Yield–Quality Relationships

**Figure 2:**
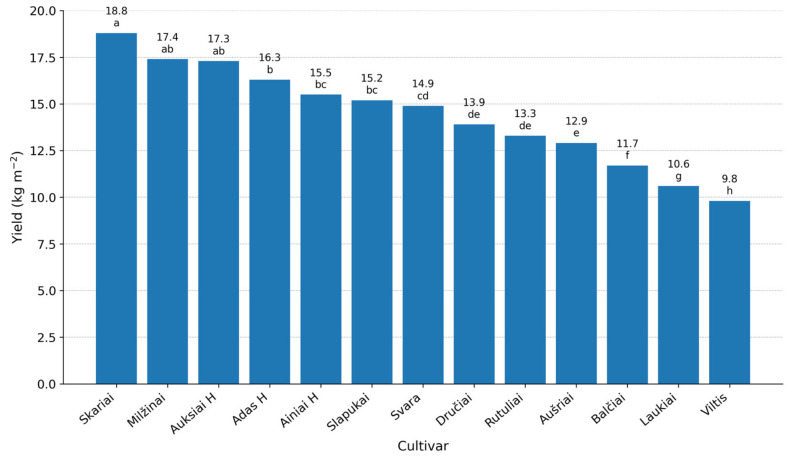
Average yield of Lithuanian tomato cultivars and hybrids grown in natural soil under unheated greenhouse conditions (data adapted from Visockiene [10], Radzevičius [13] and Radzevicius et al. [29]). Different letters indicate significant differences at *p* ≤ 0.05.

Published cultivar comparison data indicate that the highest yields were recorded in ‘Skariai’ (18.8 kg m^−2^), followed by ‘Milžinai’ (17.4 kg m^−2^) and the hybrid ‘Auksiai H’ (17.3 kg m^−2^) (Figure 2). These genotypes clearly represent a high-performance group characterized by strong production potential. Similarly, other hybrids, including ‘Adas H’ (16.3 kg m^−2^) and ‘Ainiai H’ (15.5 kg m^−2^), also ranked among the higher-yielding entries, confirming the contribution of heterosis to increased productivity.

In contrast, the lowest yield values were observed in ‘Viltis’ (9.8 kg m^−2^) and ‘Laukiai’ (10.6 kg m^−2^), indicating limited productivity under the tested conditions. A broad group of cultivars (12–15 kg m^−2^), including ‘Aušriai’, ‘Dručiai’, ‘Rutuliai’, ‘Svara’, and ‘Balčiai’, demonstrated intermediate and relatively stable yield performance.

The highest yield observed in cultivars such as ‘Skariai’, ‘Milžinai’, and hybrid ‘Auksiai H’ shows the significant role of genetic background in determining productivity. In particular, the superior performance of hybrid genotypes (e.g., ‘Auksiai H’, ‘Adas H’, ‘Ainiai H’) confirms the well-established contribution of heterosis (hybrid vigor) to increased yield in tomato, resulting from enhanced biomass accumulation, improved resource-use efficiency, and greater reproductive capacity [13,44,45,46].

Hybrid cultivars typically exhibit improved sink strength and more efficient assimilate partitioning toward fruit development, which explains their consistently higher productivity compared to open-pollinated varieties [13,44]. This effect is especially pronounced under controlled or greenhouse conditions, where reduced environmental constraints enable a more complete expression of genetic potential.

The lower yields observed in cultivars such as ‘Viltis’ and ‘Laukiai’ suggest either reduced sink capacity or less efficient adaptation to the specific growing conditions. However, it is important to note that lower-yielding genotypes may still possess desirable traits such as enhanced nutritional quality or stress tolerance, which are often negatively correlated with yield [12,13,14].

For comparison, tomato yields under low-input cultivation systems in Europe vary widely depending on region, season length, greenhouse type, and management intensity. In Mediterranean low-input or unheated greenhouse systems, reported tomato yields commonly range from approximately 11 to 19 kg m^−2^, with values of 11.0–14.5 kg m^−2^ reported for spring cultivation and approximately 19.0 kg m^−2^ under minimally heated winter greenhouse conditions [81,82]. In Spain, conventional greenhouse production without intensive technological inputs has also been reported to produce approximately 11.5–12.2 kg m^−2^ [83]. Within this context, the yield range observed among Lithuanian cultivars grown in unheated greenhouses (9.8–18.8 kg m^−2^) is comparable with values reported for non-intensive Mediterranean protected cultivation systems. Notably, ‘Skariai’ (18.8 kg m^−2^), ‘Milžinai’ (17.4 kg m^−2^), and the hybrid ‘Auksiai H’ (17.3 kg m^−2^) reached the upper range of yields reported for low-input greenhouse production, indicating strong adaptation to temperate, low-energy cultivation conditions.

The group of cultivars showing intermediate but stable yields (12–15 kg m^−2^) is particularly relevant from a practical standpoint, as yield demonstrated greater stability is a key trait in sustainable production systems. Stability across varying environmental conditions is often associated with broader adaptability and resilience, especially in temperate climates characterized by fluctuating temperature and light regimes [35,36].

Part of this variation likely suggests dilution effects, where rapid fruit biomass accumulation reduces the concentration of soluble metabolites [12,13,14]. This relationship highlights the importance of optimizing carbon allocation between vegetative growth, fruit development, and metabolite accumulation. From a breeding perspective, the challenge lies in developing cultivars that combine high yield potential with superior fruit quality and environmental adaptability. Recent approaches emphasize the integration of physiological, genetic, and agronomic strategies to improve yield without compromising quality, including the use of molecular markers, improved hybrid combinations, and optimized cultivation practices. Overcoming this limitation requires integrating physiological understanding with molecular breeding approaches, particularly genomic selection and metabolic pathway optimization [44,45,46].

**Figure 3:**
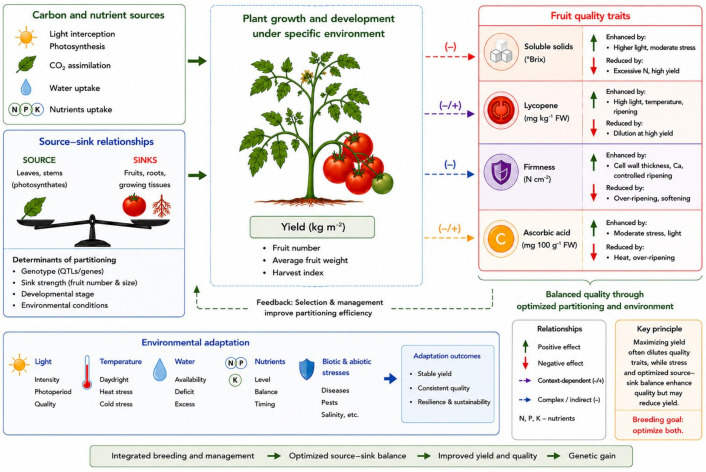
Conceptual yield–quality trade-off model linking yield, soluble solids, lycopene, firmness, source and environmental adaptation.

The available studies indicate that Lithuanian tomato germplasm includes both high-yielding hybrids and stable-performing cultivars, providing a useful basis for breeding programs aimed at improving productivity under Northern European growing conditions (Figure 3). Nevertheless, the evidence also suggests that the unresolved balance between productivity, nutritional quality, and sensory traits remains one of the main challenges for future Lithuanian tomato breeding.

## 4. Conclusions

Lithuanian tomato germplasm represents a valuable regional breeding resource characterized by moderate molecular diversity, distinct pedigree structure, and broad phenotypic variation in fruit morphology, biochemical composition, technological traits, and yield performance (Table 4). Available SSR-based data indicate that Lithuanian cultivars and hybrids possess moderate genetic variability, but also confirm a relatively narrow genetic base typical of cultivated tomato and regional breeding programs. This narrowness is not only a limitation but also reflects long-term selection for adaptation to Northern European conditions, including variable temperature, reduced light intensity, and short growing seasons.

The reviewed evidence shows that Lithuanian cultivars contain useful combinations of fruit-quality traits. Traditional cultivars frequently demonstrate higher lycopene content, good dry matter, and balanced soluble solids, whereas hybrids tend to show stronger productivity, firmness, and uniformity. Cultivars such as ‘Svara’, ‘Slapukai’, ‘Balčiai’, and ‘Milžinai’ appear particularly valuable for nutritional and technological quality, while ‘Skariai’, ‘Auksiai H’, ‘Adas H’, and ‘Ainiai H’ contribute strong yield potential. However, these trait patterns must be interpreted cautiously because available data originate from different studies, environments, and analytical methods.

A central conclusion of this review is that Lithuanian tomato breeding faces the same major challenge as global tomato improvement: combining high yield with superior flavor, nutritional quality, firmness, and environmental adaptation. The current germplasm offers promising donor material for this purpose, but further genetic gain will require broader use of molecular and genomic tools. Future studies should move beyond limited SSR characterization and apply SNP genotyping, GWAS, genomic selection, high-throughput phenotyping, metabolomics, and pangenomic approaches. Such integration would allow breeders to better connect genetic diversity with realized breeding progress.

The main value of Lithuanian tomato germplasm may therefore lie less in exceptional diversity itself and more in the stabilization of useful trait combinations under temperate production environments.

Future research should prioritize genome-wide characterization of Lithuanian tomato germplasm using SNP genotyping, pangenomic resources, and multi-environment phenotyping to enable the transition from descriptive diversity studies toward predictive breeding and measurable genetic gain.

## Figures and Tables

**Table 1:** Summary of genetic diversity and molecular characterization parameters of Lithuanian tomato cultivars and hybrids (Radzevičius et al. [32]).

Parameter	Cultivars (*n* = 13)	Hybrids (*n* = 6)	Remarks
Number of SSR markers	7	7	Same marker set used for all genotypes
Total number of alleles	24	26	Slightly higher allelic richness in hybrids
Mean alleles per locus	3.43	3.71	Indicates moderate genetic diversity
Expected heterozygosity (*He*)	0.51	0.51	*He* was calculated from allele frequencies and reflects population-level genetic diversity rather than individual heterozygosity. Similar *He* values indicate comparable allele-frequency distributions across SSR loci despite clear differences in observed zygosity between cultivars and hybrids
Observed heterozygosity (*Ho*)	0.00	0.34	Cultivars were homozygous at all analyzed SSR loci, whereas hybrids exhibited heterozygosity inherited from genetically distinct parental lines
*PIC* (mean)	0.47	0.45	Moderate marker informativeness
*PIC* range	0.13–0.68	0.31–0.61	Wider range observed in cultivars
Zygosity	Homozygous	Partially heterozygous	Reflects breeding system and hybrid origin
Genetic structure	Pedigree-based clustering	Mixed inheritance	Based on UPGMA cluster analysis
Gene pool diversity	Narrow	Slightly broader	Compared with global tomato germplasm

**Table 2:** Molecular targets and breeding relevance of key tomato traits.

Trait Group	Major Genes/QTLs/Pathways	Breeding Relevance	Molecular Approach	Representative References
Fruit size	*FW2.2*, *FW3.2*, *CSR*	Yield, market class	MAS, GWAS, genomic selection	[52,66]
Fruit shape	*SUN*, *OVATE*, *FASCIATED*	Market type, processing suitability	MAS, QTL mapping	[50,51]
Growth habit	*SP*, *SFT*	Determinate/indeterminate architecture	MAS, genome editing	[49,50,51,52]
Ripening and shelf life	*RIN*, *NOR*, *CNR*, ethylene pathway	Postharvest quality, firmness	MAS, CRISPR/Cas, transcriptomics	[11,67,70,71]
Firmness	Cell wall metabolism genes, polygalacturonase, expansins	Transportability, shelf life	MAS, transcriptomics	[67,70,71]
Lycopene and carotenoids	*PSY1*, *LCY-B*, carotenoid pathway	Nutritional quality	MAS, metabolomics, genome editing	[64,65,66,67]
Soluble solids and flavor	Sugar metabolism, organic acids, volatile pathways	Consumer acceptance	GS, metabolomics, GWAS	[7,10,59,60,61]
Yield stability	Polygenic source–sink regulation	Productivity and adaptation	GS, phenomics	[44,45,46,59,60,61,62,63]
Disease resistance	*Tm-2^2^*, *Ve1*, *I*, *I-2*, *I-3*, *Mi-1.2*, *Ph-3*, *Ty-1/Ty-3*, *Ty-2*, *Sw-5b*	Resistance to Tomato mosaic virus, Verticillium wilt, Fusarium wilt, root-knot nematodes, late blight, Tomato yellow leaf curl virus, Tomato spotted wilt virus	MAS, marker pyramiding, GWAS, genomic selection	[64,65,66]

**Table 3:** Characteristics of Lithuanian tomato cultivars and fruit quality parameters *.

Cultivar/Hybrid	Plant Type	Fruit Shape	Average Fruit Mass (g)	Dry Matter (%)	Soluble Dry Matter (°Brix)	Fruit Firmness (N cm^−2^)	Lycopene (mg kg^−1^ FW)	Ascorbic Acid (mg 100 g^−1^ FW)
Aušriai	determinant	slightly flat	89	7.2	5.7	17.8	80	14.1
Viltis	determinant	slightly flat	113	6.5	5.5	15.5	86	7.8
Laukiai	determinant	slightly flat	64	6.1	5.7	15.2	89	12.2
Skariai	indeterminate	cylindrical	138	7.0	6.0	12.5	73	12.6
Slapukai	determinant	slightly flat	95	7.5	6.5	17.7	83	19.0
Dručiai	indeterminate	slightly flat	77	4.8	4.7	13.2	84	9.4
Milžinai	indeterminate	slightly flat	161	7.0	5.6	14.6	91	7.9
Rutuliai	indeterminate	slightly flat	101	6.3	5.7	13.6	72	7.8
Svara	determinant	round	60	7.3	5.6	18.2	95	10.9
Balčiai	determinant	slightly flat	60	7.4	5.9	22.9	78	19.3
Auksiai H	indeterminate	round	38	4.6	4.3	19.1	38.4	17.6
Adas H	indeterminate	round	35	8.1	6.1	20.3	40.3	17.4
Ainiai H	determinant	obovoid	67	6.39	5.52	16.3	71.4	17.8

* Data were compiled from Lithuanian studies conducted in Lithuania, where tomatoes were grown in natural soil under unheated greenhouse conditions according to the standardized tomato cultivation technology adopted by the Institute of Horticulture [78]. The data summarized in this table should be interpreted within a common Lithuanian cultivation framework. Because all evaluated cultivars and hybrids were grown using the same cultivation technology under comparable unheated greenhouse conditions, the reported values provide a meaningful basis for cultivar-level comparisons. Nevertheless, fruit quality traits such as soluble solids, carotenoids, ascorbic acid, and dry matter remain sensitive to year-to-year variation in light intensity, temperature, and water availability. Therefore, absolute values should not be interpreted as results originating from a single uniform multi-year experiment. Rather, the table is intended to identify broad cultivar-level patterns, relative differences among genotypes, and breeding-relevant tendencies under Lithuanian temperate greenhouse conditions.

**Table 4:** Strengths and limitations of Lithuanian tomato germplasm for future breeding.

Category	Strengths	Limitations	Future Breeding Priorities
Environmental adaptation	Adapted to Northern European conditions, including fluctuating temperatures and lower light intensity	Limited evaluation under diverse climatic conditions	Multi-environment trials and genotype × environment analysis
Nutritional quality	High lycopene, balanced soluble solids, good dry matter, and elevated ascorbic acid in several traditional cultivars	Nutritional traits are not always associated with high productivity	Combine nutritional quality with yield and shelf-life stability
Fruit morphology	Diversity in fruit size, shape, and plant growth type supports multiple market classes	Diversity remains narrower than in global germplasm collections	Expand breeding pools and diversify trait combinations
Yield performance	Several hybrids and cultivars demonstrate stable productivity under greenhouse conditions	Persistent yield–quality trade-offs	Improve simultaneous selection for yield and fruit quality
Postharvest traits	Some cultivars show excellent firmness and transportability	Increased firmness may reduce sensory quality	Balance postharvest performance with eating quality
Genetic diversity	Moderate SSR-based diversity and coherent pedigree structure	Relatively narrow breeding base and limited allelic richness	Broaden germplasm through introgression and international material
Local breeding value	Long-term regional selection stabilized useful trait combinations	Risk of limited adaptive potential under future climate change	Integrate local adaptation with broader genetic resources
Molecular characterization	Existing SSR studies provide initial genetic framework	Lack of high-resolution genomic characterization	Apply SNP genotyping, GWAS, genomic selection, and pangenomics
Modern breeding potential	Suitable material for genomic selection and quality-oriented breeding	Limited integration of omics and high-throughput phenotyping	Combine conventional breeding with genomics and phenomics
Strategic importance	Valuable regional donor material for temperate-environment breeding	Not exceptionally diverse on a global scale	Use as complementary germplasm within broader breeding programs

## Data Availability

No new data were created or analyzed in this study. Data sharing is not applicable to this article.

## Supplementary Materials

The following supporting information can be downloaded at: https://www.mdpi.com/article/10.3390/ijms27125433/s1.
