# Supplementary figures and images for: From Genetic Diversity to Genetic Gain: Molecular Approaches and Breeding Strategies in Tomato with Insights from Lithuanian Germplasm
Source: Int J Mol Sci. 2026 Jun 16;27(12):5433. doi: 10.3390/ijms27125433 (PMC13300719; doi:10.3390/ijms27125433)

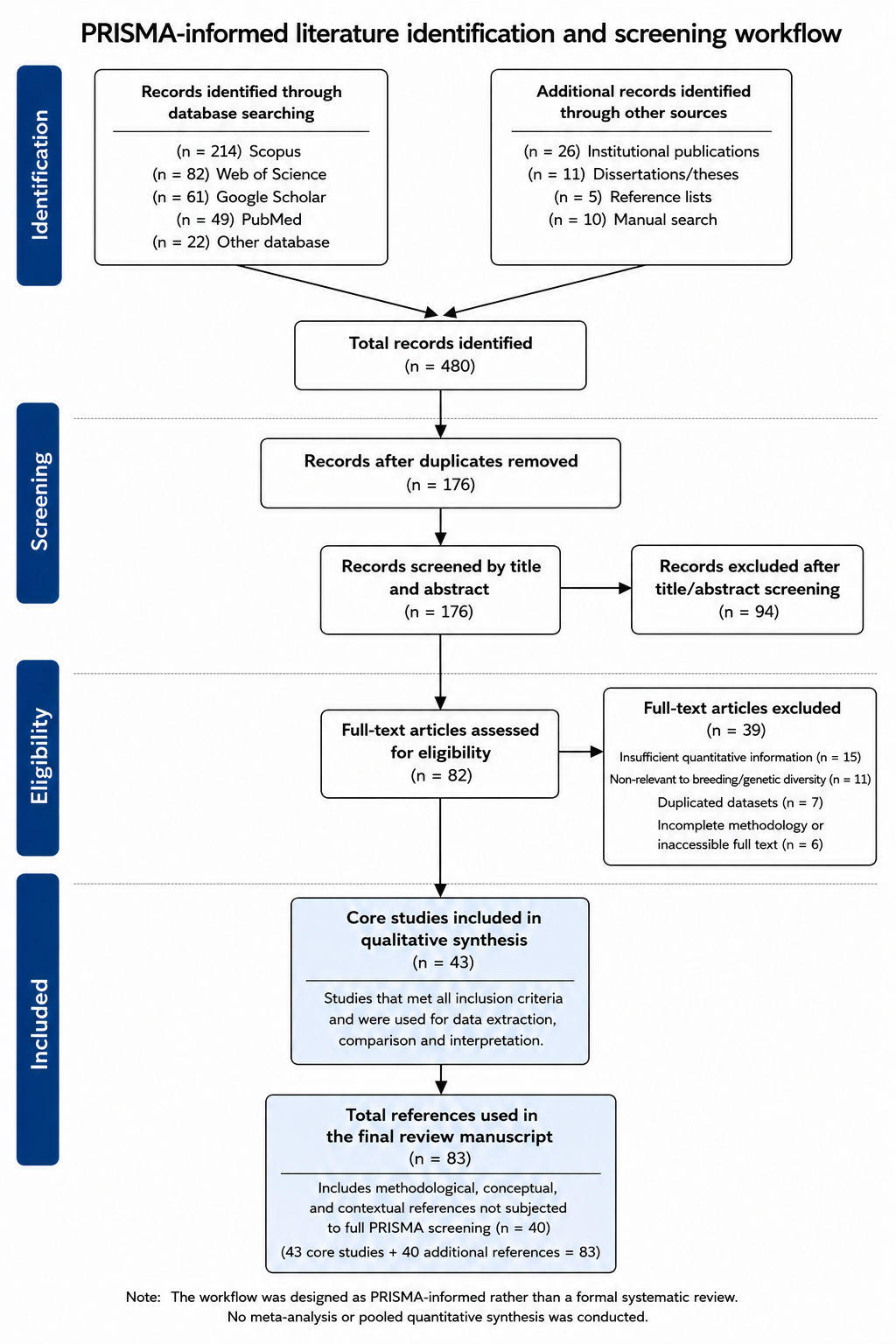

Supplement: Supplementary file 1 [file ijms-27-05433-s001.zip › ijms-4338979-supplementary.png]
